# Non-myopic multipoint multifidelity Bayesian framework for multidisciplinary design

**DOI:** 10.1038/s41598-023-48757-3

**Published:** 2023-12-18

**Authors:** Francesco Di Fiore, Laura Mainini

**Affiliations:** 1 Departement of Mechanical and Aerospace Engineering, Politecnico di Torino, 10129 Turin, Italy; 2 Department of Aeronautics, Imperial College London, London, SW7 2AZ UK

**Keywords:** Aerospace engineering, Applied mathematics, Computational science, Computer science

## Abstract

The adoption of high-fidelity models in multidisciplinary design optimization (MDO) permits to enhance the identification of superior design configurations, but would prohibitively rise the demand for computational resources and time. Multifidelity Bayesian Optimization (MFBO) efficiently combines information from multiple models at different levels of fidelity to accelerate the MDO procedure. State-of-the-art MFBO methods currently meet two major limitations: (i) the sequential adaptive sampling precludes parallel computations of high-fidelity models, and (ii) the search scheme measures the utility of new design evaluations only at the immediate next iteration. This paper proposes a Non-Myopic Multipoint Multifidelity Bayesian Optimization (NM3-BO) algorithm to sensitively accelerate MDO overcoming the limitations of standard methods. NM3-BO selects a batch of promising design configurations to be evaluated in parallel, and quantifies the expected long-term improvement of these designs at future steps of the optimization. Our learning scheme leverages an original acquisition function based on the combination of a two-step lookahead policy and a local penalization strategy to measure the future utility achieved evaluating multiple design configurations simultaneously. We observe that the proposed framework permits to sensitively accelerate the MDO of a space vehicle and outperforms popular algorithms.

## Introduction

Optimization is becoming essential in science and engineering to empower the performance and sustainability of complex systems toward global challenges, such as green development and climate change. In many real-world applications, the optimization of advanced technologies involves multiple scientific disciplines characterized by complex relationships and couplings difficult to be tackled. Multidisciplinary design optimization (MDO) relates to the development of computational methodologies for the design and optimization of complex systems taking into account the interactions of multiple disciplines^1,2^. Those interactions can span different strength and width of the cross disciplinary couplings^3,4^. MDO approaches have been applied to the design and optimization of a broad range of engineering systems including aircraft^5^, spacecrafts^6^, launch vehicles^7^, buildings^8^, electric automobiles^9^, ships^10^, energy systems^11^ and robots^12^. One of the major challenges addressed by MDO is represented by the possibility to use expensive high-fidelity disciplinary models, such as the ones that are given by large scale systems of equations for the numerical solutions of partially differential equations, directly in the simulation-based optimization process^13,14^. Indeed, the search of optimal design solutions would benefit from accurate representations of the system behaviour and physics including the couplings across the multiple disciplines. However, considering all those interactions and complex couplings would typically demand for large amount of evaluations of the high-fidelity disciplinary representations which would result in prohibitive computational costs for the overall MDO procedure.

To address these challenges, MDO literature proposed a variety of solutions that rely on the use of low-fidelity models to reduce the computational burden and complexity associated with disciplinary analysis and save computing resources. As discussed by Peherstorfer et al.^15^, low-fidelity disciplinary solvers range from simplified models directly derived from the high-fidelity counterpart using expert knowledge^16^, to projection-based models that identify a low-fidelity subspace retaining the essential features of the system^17^, and to surrogate-based models where the input-output relationships of disciplines are derived from observations of the high-fidelity model^18^. Even if the evaluation of these low-fidelity representations could be sensitively reduced in computational cost, the former simplified models might not be adequate to depict complex non-linear phenomena that frequently characterize the disciplinary domain, while the latter projection-based and surrogate-based models might require a large amount of costly high-fidelity data for their construction.

Multifidelity methods acknowledge the opportunity offered by low-fidelity representations and offer approaches to address the research gap of including expensive high-fidelity disciplinary analysis into the MDO process^19^. Multifidelity methods combines data extracted from a library of disciplinary models that can be hierarchically ordered according to accuracy and computational cost^15,20^. The availability of multiple levels of fidelity can be exploited to support the search procedure through a principled elicitation of information: fast low-fidelity models are used to massively explore different design configurations, and expensive high-fidelity models sparingly refine the solution of the MDO problem. Multifidelity methods have been successfully applied to a variety of MDO applications ranging from aircraft^21,22^ and space vehicles^23,24^ to ships^25^ and unmanned underwater vehicles^26^, from electric^27^ and hybrid^28^ vehicles to green energy technologies^29,30^. In most cases, the complexity of the disciplinary analysis and couplings discourages the use of gradient-based optimization strategies: the computation of the derivatives might demand for massive high-fidelity data and increase the overall computational burden. Therefore, the MDO procedure commonly relies on a black-box approach where the disciplinary analyses are regarded as a pure input/output relationship, whose information about the mathematical properties and derivatives are not available.

Multifidelity Bayesian optimization (MFBO) provides a computational framework for black-box optimization and leverages disciplinary solvers at different levels of fidelity to accelerate the identification of promising design solutions^31–33^. MFBO realizes an adaptive sampling scheme based on a multifidelity acquisition function that targets the design improvement with a continuous trade-off between optimization performance and computational cost. Several MFBO approaches have been proposed to address a variety of design problems. Charayron et al.^34^ applied an original multifidelity Bayesian framework for the MDO of a full electric drone accounting for a long-range surveillance mission. Serani et al.^35^ proposed a MFBO based on stochastic radial-basis functions surrogate applied to the design optimization of a destroyer-type vessel. Reisenthel et al.^36^ adopted an MFBO framework for the aeroelastic design optimization of a UAV wing. Tran et al.^37^ implemented MFBO for the chemical design optimization of atomistic materials to identify the optimal bulk modulus.

Most state-of-the-art MFBO algorithms are greedy and sequential in nature since the multifidelity acquisition function (i) quantifies only how the design and level of fidelity selected at the current iteration affect the immediate next step, and (ii) selects only a single combination of design variables and level of fidelity to be evaluated at the next iteration. In particular, (i) the popular greedy approach precludes greater informative gains that can be acquired through the measure of the long-term reward obtained at future steps of the optimization, and might preclude superior accelerations of the MDO procedure; (ii) the sequential search might not be computationally efficient for MDO problems where the simulation of complex interdisciplinary relationships demands for a huge amount of high-fidelity data and associated computational expense.

To address these gaps, we propose a non-myopic multipoint multifidelity Bayesian optimization (NM3-BO) framework that aims to overcome both greedy and sequential limitations of standard MFBO methodologies. Our original multifidelity search defines an optimal sequence of decisions to (i) maximize the long-term reward as the improvement of the optimal design solution achieved at future steps of the optimization, and (ii) select a batch of design configurations and levels of fidelity to be evaluated simultaneously. To overcome the shortcomings of standard MFBO, our non-myopic multipoint and multifidelity learning scheme (i) is derived formalizing MFBO as a dynamic system under uncertainty addressed through a dynamic programming technique, (ii) defines an optimal policy as a sequence of decisions to maximize the two steps ahead utility obtained evaluating a design with a specific disciplinary model, and (iii) uses a local penalization strategy to enable multiple decisions as a batch of paired designs and levels of fidelity to query in parallel.

The performance of the proposed NM3-BO are illustrated and discussed in comparison with standard MFBO frameworks for the multidisciplinary design optimization of a re-entry vehicle. We adopt the Multidisciplinary Feasible (MDF) formulation to formalize the space-vehicle MDO problem, and capture the multidisciplinary nature of the system considering the contributions of the propulsion system, re-entry descend trajectory, aerothermodynamic effects, and thermo-structural interactions. In particular, the specific MDF architecture considers the coupling between the trajectory and the aerothermodynamic disciplines through the aerodynamic coefficients, and the coupling between the thermo-structural and aerothermodynamic disciplines through the thermal protection system wall temperature. This design problem is specifically selected to exemplify the marked cross-disciplinary scenario and strong couplings between disciplines that can be traced in the vast majority of MDO problems in science and engineering.

## Methodology

The non-myopic multipoint multifidelity Bayesian optimization (NM3-BO) framework formalizes an adaptive sampling scheme that measures the long-term utility of a batch of design configurations evaluated simultaneously. Our formulation regards MFBO as a decision making problem affected by uncertainty: the decision task relates to the selection of promising design configurations – combination of design parameters – and the associated levels of fidelity to query; the uncertainty elements relate to the black-box nature of the objective function and the probabilistic prediction of the surrogate model.

In the following, the MDO problem setup and the BO single-fidelity and multifidelity frameworks are firstly introduced to provide an overview of the core background of our work. Then, we formalize the optimal policy for MFBO and illustrate how to robustly approximate it through a Monte Carlo technique. In addition, we propose a multiple decision making strategy to enable parallel computations of a batch of designs and associated levels of fidelity. Finally, the NM3-BO algorithmic framework is presented and discussed.

### MDO problem setup

This work considers the general formulation of the MDO problem as follows^4^:

1 $$\begin{aligned} \begin{aligned} \text {minimize} \quad&f({\textbf {x}}) \\ \text {with respect to} \quad&{\textbf {x}}\\ \quad \text {subject to} \quad&c({\textbf {x}}) \ge 0 \\ \quad \quad&\mathscr {R}_{i}({\textbf {x}}) = 0 \quad \text {for} \; i= 1,...,D\end{aligned} \end{aligned}$$

where the goal is to identify a set of design variables ${\textbf {x}}$ that minimizes an objective function $f$ subject to design constraints $c$, and the solution of governing equations in residual form $\mathscr {R}_{i}({\textbf {x}})$ for each $i$-th discipline.

The disciplinary analyses are usually performed through black-box simulations: computer codes operate independently and define relationships between inputs and outputs, and hide the procedure associated with their computation. High-fidelity disciplinary analyses involve the numerical solution of governing PDEs through expensive computational procedures, such as Computational Fluid Dynamics (CFD) techniques for the numerical solution of Navier-Stokes equations.

### BO from single-fidelity to multifidelity

Bayesian optimization (BO) is an efficient computational strategy to address the MDO of expensive black-box objective functions^38,39^. To solve Eq. (1), BO uses two key components: a surrogate model of the objective function $f({\textbf {x}})$ and an acquisition function computed on the surrogate of $f$. The maximization of the acquisition function permits to select the most promising design configuration ${\textbf {x}}'$ to query. BO uses the observed value $f({\textbf {x}}')$ to update the surrogate model and the process iterates until a certain termination criteria is reached. Popular formulations of Bayesian optimization and acquisition functions are overviewed by Frazier et al.^39^ and Shahriari et al.^38^.

The most widely used acquisition functions determine a greedy and sequential adaptive sampling scheme that considers only the immediate effect of evaluating the objective function for a single design, and do not consider the potential gains introduced in future evaluations. To address this type of greedy limitation, BO has been formalized as a partially observable Markov decision process^40^, and several works^41–44^ provide solutions to this process and formalize non-myopic multifidelity acquisition functions. In addition, multipoint formulations of the BO framework have been proposed to evaluate in parallel multiple designs with a single level of fidelity of the disciplinary model^45–47^. However, the combination of non-myopic and multipoint formulations in literature are conceived exclusively for a single-fidelity framework only: the optimization process relies on the responses of disciplinary models at one single fixed level of fidelity. In the MDO context, this single fidelity approach could hinder the expensive high-fidelity disciplinary models to be interrogated directly during the search, which otherwise would result in prohibitive computational costs. In addition, the computational cost becomes unmanageable as it scales exponentially when the disciplinary couplings are also considered during the process: the identification of an optimal design that satisfies all the interactions and couplings across the disciplines would require massive evaluations of high-fidelity disciplinary models with the associated growth of the computational demand.

In many applications, scientists and engineers might rely on different disciplinary models of the objective function and constraints with different degrees of accuracy and associated demand for computational resources. Multifidelity Bayesian Optimization (MFBO) combines disciplinary responses from this library of models $\{f^{(1)},f^{(2)},...,f^{(L)} \}$ hierarchically ordered according to the level of fidelity $l= 1,...,L$ to accelerate the solution of the MDO problem (Eq. 1). In this setting, the surrogate model synthesizes the outcomes computed with multiple models into a unique surrogate, and the acquisition function defines an adaptive sampling scheme that identifies the design configuration and the associated level of fidelity to query at each iteration.

#### Multifidelity Gaussian process

We adopt the Multifidelity Gaussian process (MFGP) as the surrogate model of the objective function given the successful application over a variety of methodologies and applications^47–49^. MFGP is formalized extending the Gaussian process^50^ (GP) formulation to multiple levels of fidelity, and provides a prediction of the objective function over the design space $\mathscr {X}$ through the mean function $\mu ^{(l)}({\textbf {x}}) = \mathbb {E}[f^{(l)}({\textbf {x}})]$, and the associated uncertainty of the prediction $\sigma ^{2(l)}({\textbf {x}}) = \mathbb {E}[(f^{(l)}({\textbf {x}})-\mu ({\textbf {x}}))(f^{(l)}({\textbf {x}}')-\mu ({\textbf {x}}'))]$ through a covariance function.

MFGP relies on an autoregressive scheme to synthesize responses from models at different levels of fidelity^51^:

2 $$\begin{aligned} f^{(l)} = \rho _{l-1} f^{(l- 1)} \left( {\textbf {x}}\right) + \delta ^{(l)} \left( {\textbf {x}}\right) \quad l= 2,...,L\end{aligned}$$

where $\rho _{l-1}$ is a regression parameter that scales successive representations $f^{(l)}$ and $f^{(l- 1)}$, and $\delta ^{(l)}$ models the discrepancy between $f^{(l)}$ and the scaled model $\rho _{l-1} f^{(l- 1)}$. This discrepancy term $\delta ^{(l)}$ is assumed to be a Gaussian process characterized by mean function $\upsilon ({\textbf {x}})^{T} \beta ^{(l)}$ and covariance $\kappa ^{(l)} \left( {\textbf {x}},{\textbf {x}}'\right)$, where $\upsilon$ is the vector of regression functions and $\beta ^{(l)}$ are the associated weighting coefficients. We use the Gaussian correlation model as the covariance function of the MFGP surrogate:

3 $$\begin{aligned} \kappa ({\textbf {x}}, {\textbf {x}}') = \varsigma ^2_{l} \exp \left\{ - \sum _{m=1}^{M} \varpi ^m_{l} ({\textbf {x}}_m - {\textbf {x}}_m')^2 \right\} \end{aligned}$$

where $\varpi = (\varpi ^1_{l}, \varpi ^2_{l}, ..., \varpi ^M_{l})$ is the roughness parameter, and $\varsigma ^2_{l}$ is the process variance of the $l$-th level of fidelity.

Thus, the posterior mean $\mu ^{(l)} ({\textbf {x}})$ and variance $\sigma ^{2(l)} ({\textbf {x}})$ of the objective are formalized through the covariance matrix ${\textbf {K}}(i, j) = \left[ \kappa \left( \left( {\textbf {x}}_{i}, l_i \right) , \left( {\textbf {x}}_{j}, l_j \right) \right) + \gamma _{ij}\sigma _{\epsilon }({\textbf {x}}_i) \right]$ and $\kappa _N^{(l)} ({\textbf {x}}) = \left[ \kappa \left( \left( {\textbf {x}}_{i}, l_i \right) , \left( {\textbf {x}}, l\right) \right) \right]$:

4 $$\begin{aligned}{} & {} \mu ^{(l)} ({\textbf {x}}) = \kappa _N^{(l)} ({\textbf {x}})^T {\textbf {K}}^{-1} {\textbf {y}} \end{aligned}$$

5 $$\begin{aligned}{} & {} \sigma ^{2(l)} ({\textbf {x}}) = \kappa \left( \left( {\textbf {x}}, l\right) , \left( {\textbf {x}}, l\right) \right) - \kappa _N^{(l)} ({\textbf {x}})^T {\textbf {K}}^{-1} \kappa _N^{(l)} ({\textbf {x}}) \end{aligned}$$

where $\gamma _{ij}$ is the Kronecker delta function. The hyperparameters $(\rho , \beta , \varpi , \varsigma )$ of the multifidelity Gaussian process surrogate model are estimated through maximum likelihood estimation methodology^52^.

#### Multifidelity expected improvement

The multifidelity acquisition function $U$ quantifies the utility of new design configurations with a trade-off between cost ad accuracy of the associated model to query. In this work, we will build our method onto the Multifidelity Expected Improvement (MFEI) acquisition function^31^, given the popularity of this formulation across different science and engineering communities and the variety of adoptions documented in literature^36,53^. The MFEI is formulated as follows:

6 $$\begin{aligned} U_{MFEI}({\textbf {x}}, l) = U_{EI}({\textbf {x}}) \alpha _1({\textbf {x}},l) \alpha _2({\textbf {x}},l) \alpha _3(l) \end{aligned}$$

where $U_{EI}$ is the expected improvement acquisition function computed with the highest level of fidelity. The utility functions $\alpha _1, \alpha _2$ and $\alpha _3$ are defined as follows:

7 $$\begin{aligned}{} & {} \alpha _1({\textbf {x}},l) = corr[f^{(l)}({\textbf {x}}),f^{(L)}({\textbf {x}})] \end{aligned}$$

8 $$\begin{aligned}{} & {} \alpha _2({\textbf {x}},l) = 1- \frac{\sigma _{\epsilon }}{\sqrt{\sigma ^{2(l)}(\textbf {x})-\sigma _{\epsilon }^2}} \end{aligned}$$

9 $$\begin{aligned}{} & {} \alpha _3(l) = \frac{\lambda ^{(L)}}{\lambda ^{(l)}}. \end{aligned}$$

$\alpha _1({\textbf {x}},l)$ reflects the decrease of accuracy associated with lower-fidelities quantified as the correlation between the $l$-th fidelity outcome $f^{(l)}({\textbf {x}})$ and the high-fidelity $f^{(L)}({\textbf {x}})$ disciplinary analysis. $\alpha _2({\textbf {x}},l)$ brings awareness about the stochastic nature of the objective function through the measurement noise $\sigma _{\epsilon }$, and considers the reduction of the uncertainty associated with the evaluation of design solutions with the $l$-th model. $\alpha _3(l)$ includes the computational cost $\lambda ^{(l)}$ of the $l$-th disciplinary analysis in the sampling procedure, and balances the accuracy with the resources entailed for the objective evaluation.

Alternative formulations of the multifidelity acquisition function have been proposed in literature, such as the Multifidelity Max-Value Entropy Search (MFMES)^32^ and the Multifidelity Probability of Improvement^33^ (MFPI). Both these acquisition functions define a sampling scheme sequential and greedy, where the design improvement is measured as the probability of lower the objective function according to the surrogate prediction (MFPI) or as the maximum decrease of differential entropy (MFMES). In this work, the proposed NM3-BO framework is compared against all those formulations of the acquisition functions for the MDO problem of a re-entry vehicle.

### Optimal decision making process over the next two-step ahead

The multifidelity Bayesian optimization is regarded as a decision making problem and formulated as a Markov Decision Process (MDP). MDPs are discrete-time stochastic control processes that allow to model the sequential decision making process of a dynamic system under uncertainty^54,55^. The main methodological elements of MDPs are: (i) the Markov chains model the transitions of the dynamic system from the initial state to future states, (ii) a decision-making model makes a decision at each state transition of the system, and (iii) an utility function quantifies the reward achieved by a certain decision with reference to the given goal. The objective of MDPs is to identify the optimal set of decision to efficiently reach the given goal over time. Following the perspective of MFBO as a dynamic system under uncertainty, (i) the multifidelity Gaussian process predicts the transitions of the MFBO system to future states given the initial conditions, (ii) the MFBO system evolves making a decision at each transition on the next design and level of fidelity to be evaluated, (iii) the reward of each decision is measured through the multifidelity acquisition function that evaluates the benefits of each decision through a trade-off between obtaining utility from the current state and altering the opportunities to obtain utility in the future.

The solution of the MFBO Markov Decision Process requires a procedure to perform statistical inference on the system behaviour, and depict the transitions of the system from one state to the other after exploring every possible decision. We employ the dynamic programming^56,57^ approach to solve this specific MDP. Dynamic programming (DP) partitions the optimization procedure in simpler sub-problems defined in a recursive way across several transitions. This permits to define the optimal policy as a sequence of rules that maximizes the cumulative reward achieved making decisions at future iterations. In the following, we formulate the optimization policy based on an original multifidelity acquisition function to measure the utility of decisions expected over future steps of the optimization procedure.

Let us consider the MFBO dynamic system fully characterized at each time step $t$ by a state $s_{t} \in \mathscr {S}_{t}$, where $\mathscr {S}_{t}$ denotes a set of states that represent all the possible configurations of the system at each time step. Following the dynamic programming approach, we consider any future iteration of the optimization process as time steps $\{t,...,T\}$ of the MFBO dynamics. For a generic time step $t$, the multifidelity Gaussian process conditioned on $\mathscr {D}_{t}=\{{\textbf {x}}_{n},y^{(l_{n})},l_{n} \}_{n= 1}^{N}$ determines the posterior distribution of the objective function, where ${\textbf {x}}$ is the design point and $y^{(l)}$ is the noisy observation of the objective function at the $l$-th level of fidelity. Thus, MFBO is fully characterized by a state $s_{t}$ defined through the dataset $\mathscr {D}_{t} \in \mathscr {S}_{t}$. Based on the simulated scenario defined by the surrogate model, MFBO makes an action $a_{t} = \{{\textbf {x}}_{t+1}, l_{t+1} \}$ that activates the dynamic of the system, and defines the next design ${\textbf {x}}_{t+1} \in \mathscr {X}$ and associated level of fidelity $l_{t+1}$ to evaluate. This action is taken under an optimization policy $\pi _{t}:\mathscr {S}_{t} \rightarrow \mathscr {X}$ that maximizes the utility achieved in the future steps by mapping the state $s_{t}$ to the action $a_{t}=\pi _{t}(s_{t})$.

At the new time step $t+1$, the value of $f^{(l_{t+1})}({\textbf {x}}_{t+1})$ is unknown and requires the evaluation of the objective function at the $l_{t+1}$-th level of fidelity. We can model this value as an uncertain quantity through the posterior distribution of the surrogate model conditioned on $\mathscr {D}_{t}$ at ${\textbf {x}}_{t+1}$ and level of fidelity $l_{t+1}$. This simulated outcome is defined as a random disturbance normally distributed $w_{t+1}^{(l)} \sim \mathscr {N}( \mu _{t}^{(l)} ({\textbf {x}}_{t+1}), \sigma _{t}^{2(l)} ({\textbf {x}}_{t+1}))$ specified by the mean $\mu _{t}^{(l)}$ and variance $\sigma _{t}^{2(l)}$ of the multifidelity Gaussian process. Once the outcome is simulated, the system transitions to the new state $s_{t+1}$ following its dynamics $\mathscr {F}$, which corresponds to the augmented dataset $\mathscr {D}_{t+1} = \mathscr {D}_{t} \cup \{{\textbf {x}}_{t+1},y^{(l_{t+1})}, l_{t+1}\}$:

10 $$\begin{aligned} \mathscr {D}_{t+1}=\mathscr {F}({\textbf {x}}_{t+1},y^{(l_{t+1})}, l_{t+1}, \mathscr {D}_{t}) \end{aligned}$$

At this point, we need to define a specific reward function for MFBO that measures the utility obtained from a simulated outcome $w_{t+1}^{(l)}$ when the action $a_{t}$ is applied to the state $s_{t}$. This reward function can be formulated as the reduction of the objective function achieved at the time step $t+1$ with respect to $t$:

11 $$\begin{aligned} r_{t}({\textbf {x}}_{t+1},y^{(l_{t+1})}, l_{t+1}, \mathscr {D}_{t}) = (f_{t}^{*(L)} - f_{t+1}^{(L)})^{+} \end{aligned}$$

where $f_{t+1}^{(L)}=w_{t+1}^{(L)}$, and $f_{t}^{*(L)}$ is the minimum value of the objective function at the highest level of fidelity observed up to $t$. Thus, we follow the DP recursive strategy and define the expected reward at the generic time step $t$:

12 $$\begin{aligned} \begin{aligned} J^{\varvec{\pi }}_{t} ({\textbf {x}}_{t+1}, l_{t+1}, \mathscr {D}_{t}) = \mathbb {E}[ r_{t} ({\textbf {x}}_{t+1},y^{(l_{t+1})}, l_{t+1}, \mathscr {D}_{t})+J_{t+1}(\mathscr {F}({\textbf {x}}_{t+1},y^{(l_{t+1})}, l_{t+1}, \mathscr {D}_{t}))] \end{aligned} \end{aligned}$$

where $\mathbb {E}\left[ r_{t}(\cdot ) \right] = U_{MFEI} ({\textbf {x}}_{t+1}, l_{t+1})$ is the multifidelity expected improvement (Eq. 6), and $J_{t+1} (\mathscr {F}( \cdot ))$ is the long-term expected reward. We formulate the two-step lookahead multifidelity acquisition function through an optimal policy $\varvec{\pi }^*$ that maximizes the cumulative expected reward over two-step ahead of a pair of design configurations ${\textbf {x}}_{t+2}$ and level of fidelity $l_{t+2}$:

13 $$\begin{aligned} \begin{aligned} U^{\varvec{\pi }^{*}}_{t}({\textbf {x}}_{t+2},l_{t+2}&, \mathscr {D}_{t+1}) = U_{MFEI} ({\textbf {x}}_{t+1}, l_{t+1}) + \mathbb {E}\left[ \max (U_{MFEI}({\textbf {x}}_{t+2},l_{t+2}))\right] \end{aligned} \end{aligned}$$

where we define the long term reward $J_{t+1} = \max (U_{MFEI}({\textbf {x}}_{t+2}, l_{t+2}))$ as the maximum of the multifidelity expected improvement conditioned on the dataset $\mathscr {D}_{t+1}$.

### Robust approximation of the optimal decision making process

The evaluation of $U^{\varvec{\pi }^{*}}_{t}$ (Eq. 13) requires the solution of nested expectations and maximizations computationally intractable. We adopt the Monte Carlo approach to avoid nested computations, and provide a robust estimate of the two-step lookahead multifidelity utility function. Let us formulate the observation of the objective function at the new design configuration ${\textbf {x}}_{t+1}$ and level of fidelity $l_{t+1}$ using the reparameterization strategy proposed by Wilson et al.^58^:

14 $$\begin{aligned} f^{(l)} \left( {\textbf {x}}_{t+1} \right) = \mu ^{(l)}_{t} + {\textbf {C}}^{(l)}_{t} \left( {\textbf {x}}_{t+1} \right) Z\end{aligned}$$

where ${\textbf {C}}^{(l)}_{t}$ is the Cholesky decomposition of the covariance matrix ${{\textbf {K}}}_{t}$, and $Z$ is an independent standard normal random variable. We use Eq. (14) to compute the mean and variance of the multifidelity Gaussian process at $t+1$ for the generic design configuration ${\textbf {x}}$:

15 $$\begin{aligned}{} & {} \mu ^{(l)}_{t+1}({\textbf {x}}) = \mu ^{(l)}_{t}({\textbf {x}}) + {\textbf {H}}^{(l)}_{t} \left( {\textbf {x}}\right) Z\end{aligned}$$

16 $$\begin{aligned}{} & {} \sigma ^{(l)}_{t+1}({\textbf {x}}) = \sigma ^{(l)}_{t}({\textbf {x}}) - {\textbf {H}}^{(l)}_{t} \left( {\textbf {x}}\right) {\textbf {H}}^{(l)}_{t} \left( {\textbf {x}}\right) ^T \end{aligned}$$

where $${\textbf {H}}^{(l)}_{t} \left( {\textbf {x}}\right) = \kappa ^{(l)}_{t} \left( {\textbf {x}}\right) {\textbf {C}}^{(l)-1}_{t}({\textbf {x}})$$.

The expectation term of our acquisition function in Eq. (13) is approximated through the prediction $\mu ^{(l)}_{t+1}$ and associated uncertainty $\sigma ^{(l)}_{t+1}$ of the multifidelity Gaussian process. This permits to estimate the multifidelity expected improvement at $t+2$ as follows:

17 $$\begin{aligned} \begin{aligned} U_{MFEI}({\textbf {x}}_{t+2}, l_{t+2}) \sim \widehat{U}_{MFEI}({\textbf {x}}_{t+2},l_{t+2},Z) \end{aligned} \end{aligned}$$

At this point, the two-step lookahead multifidelity acquisition function is evaluated sampling the random variable $Z$ and averaging many realizations of $\widehat{U}^{\varvec{\pi }^{*}}_{t}$ through Eq. (17):

18 $$\begin{aligned} \begin{aligned} U^{\varvec{\pi }^{*}}_{t}({\textbf {x}}_{t+2},l_{t+2}&, \mathscr {D}_{t+1}) \sim \mathbb {E}[\widehat{U}^{\varvec{\pi }^{*}}_{t}({\textbf {x}}_{t+2},l_{t+2}, Z)] \end{aligned} \end{aligned}$$

This Monte Carlo approach demands for a significant number of realizations of $\widehat{U}^{\varvec{\pi }^{*}}_{t}$ – in the order of thousands – to provide a robust approximation of $U^{\varvec{\pi }^{*}}_{t}$. However, we emphasize that the evaluation of Eqs. (15) and (16) is inexpensive and requires contained resources. This results in a total computational cost required by the Monte Carlo procedure that is negligible if compared to the cost associated with the evaluation of the objective function through high-fidelity disciplinary analyses in complex MDO problems.

### Enabling multiple decisions

Equation (13) provides a non-myopic multifidelity sampling scheme that sequentially selects the design configuration and the respective model to maximize the cumulative reward over two steps ahead. Di Fiore and Mainini^59^ provided evidence that this strategy permits to achieve remarkable results over a variety of benchmark analytical problems with a reduction of the number of high fidelity interrogations. However, complex multidisciplinary design optimization problems in the form of Eq. (1) open major challenges for the intrinsic demand to scale: the required accurate evaluations of the objective function can dramatically upscale during the search for improved design solutions.

To address this complex multiphysics optimization scenario, we extend the optimal policy $\varvec{\pi }^{*}$ to enable multiple decisions. This permits to define a decision making procedure that iteratively selects a batch of promising design points and associated levels of fidelity $\mathscr {B}_{i}^{n_b} = [({\textbf {x}}_{i,1},l_{i,1}),...,({\textbf {x}}_{i,n_b},l_{i,n_b})]$ while improving the design solutions over future iterations. The potential of multipoint formulations has been illustrated by^60^ for a greedy single-fidelity Bayesian framework, and motivates our proposal of a multipoint sampling strategy for the non-myopic MFBO in multidisciplinary settings. Accordingly, the formulation of our acquisition function $U^{\varvec{\pi }^{*}}_{t}$ is modified through a local penalization maximization as follows:

19 $$\begin{aligned} {\textbf {x}}_{i,k},l_{i,k} = {{\,\mathrm{arg\,max}\,}}\left[ U^{\varvec{\pi }^{*}}_{t}({\textbf {x}}_{t+2},l_{t+2}, \mathscr {D}_{t+1}) \prod _{j=1}^{k-1} \psi ({\textbf {x}},{\textbf {x}}_{j}) \right] \end{aligned}$$

where $({\textbf {x}}_{i,k},l_{i,k}) \in \mathscr {B}_{i}^{n_b}$, and $\psi$ is the local penalty function which quantifies the probability that a design point ${\textbf {x}}$ is a potential minimum not belonging to the hypersphere $\{ {\textbf {x}}\in \mathscr {X}: || {\textbf {x}}_j-{\textbf {x}}|| \le (\hat{f}^*- f^{L}({\textbf {x}}_j))/L\}$:

20 $$\begin{aligned} \psi ({\textbf {x}},{\textbf {x}}_{j}) = \frac{1}{2} erfc \left[ \frac{1}{\sqrt{2 \sigma ^{2(L)}({\textbf {x}}_j)}} \left( L|| {\textbf {x}}_j-{\textbf {x}}|| - \hat{f}^*+ \mu ^{(L)} ({\textbf {x}}_j) \right) \right] \end{aligned}$$

where *erfc* is the complementary error function, $\hat{f}^*= \min _{{\textbf {x}}\in \mathscr {X}} \mu ^{(L)}({\textbf {x}})$ is the minimum predicted by the surrogate model, and $L= \max _{{\textbf {x}}\in \mathscr {X}} || \mu ^{(L)}_{\nabla } ({\textbf {x}})||$ is the Gaussian process Lipschitz constant^60^ defined as the maximum of the surrogate gradient. The rationale behind the formulation of $\psi$ is to locally penalize the acquisition function and create exclusion zones whose amplitude is determined by the Lipschitz constant $L$. This results in small sized exclusion zones when the values of the mean function $\mu ^{(L)}$ are closer to the predicted minimum $\hat{f}^*$. In contrast, regions of the design space where the mean function $\mu ^{(L)}$ is far from the predicted minimum $\hat{f}^*$ produce larger exclusion zones. This penalization strategy emulates a sampling procedure over multiple iterations that would have been achieved by a sequential scheme if the previous evaluations of the designs in the batch were at disposal of the learner.

In addition, we provide an adaptive batch size formulation that determines the number of designs in a batch $n_b(i) = 1 + \beta /(\sqrt{2} i)$ as a function of the optimization iterations $i$ and the initial batch size $\beta$. This strategy targets the efficient use of computational resources: the number of design evaluations increases at the beginning of the optimization to improve the knowledge of the objective function distribution over the domain, and is progressively reduced to catalyze the resources toward the analyses of optimal design solutions.

### NM3-BO algorithm

Algorithm 1 illustrates the numerical implementation of our non-myopic multipoint multifidelity Bayesian optimization (NM3-BO) scheme. The initialization of the computations requires the definition of the design space $\mathscr {X}\in \mathbb {R}^d$ according to the number of variables $d$ that characterize the design configuration ${\textbf {x}}$, together with the library of models of the objective function $\{ f^{(1)},f^{(2)},...,f^{(L)} \}$. In addition, the multifidelity Gaussian process prior $GP(0,\kappa ^{(l)}({\textbf {x}},{\textbf {x}}'))$ is defined for each level of fidelity, and represents the prior belief about the distribution of the objective function over the design space. Before the start of the iterative process, an initial subset of $N_0$ design configurations $\{{\textbf {x}}_{n}\}_{n=1}^{N_0}$ and corresponding levels of fidelity $\{ l_{n}\}_{n=1}^{N_0}$ are sampled through any design of experiments technique, and are used to compute the first observations of the objective function $\{ y^{(l_n)} \}_{n=1}^{N_0}$. These data are collected into the dataset $\mathscr {D}_0 = \{ {\textbf {x}}_{n},y^{(l_n)},l_{n} \}_{n=1}^{N_0}$ which defines the initial state of the MFBO system, and induces the posterior distribution under the prior specified by the mean $\mu ^{(l)}$ and variance $\sigma ^{2(l)}$.

**Algorithm 1:**
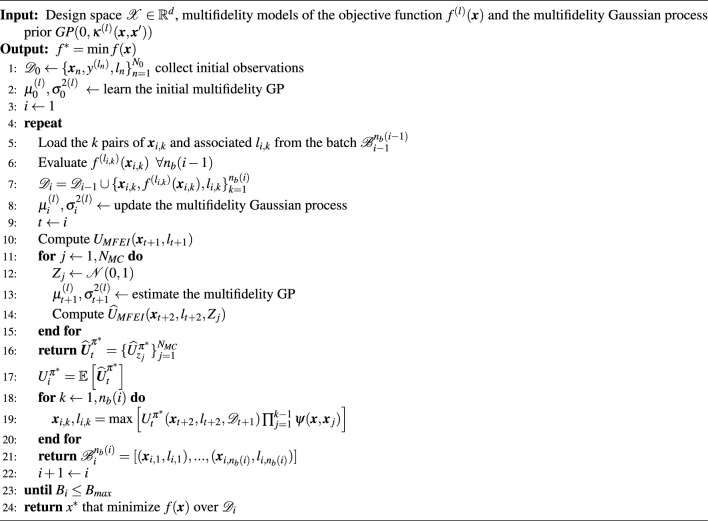
NM3-BO: non-myopic multipoint multifidelity Bayesian optimization.

For a generic iteration $i$ of the NM3-BO algorithmic flow, the surrogate model is updated through the observations of the objective function $f^{(l_{i,k})}({\textbf {x}}_{i,k})$ at each $n_b(i)$ pair of design configurations ${\textbf {x}}_{i,k}$ and levels of fidelity $l_{i,k}$ that constitutes the batch $\mathscr {B}_{i}^{n_b(i)}$ selected at the previous iteration $i-1$. This represents the new state of the MFBO system $\mathscr {D}_{i} = \mathscr {D}_{i-1} \cup \{{\textbf {x}}_{i,k},f^{(l_{i,k})}({\textbf {x}}_{i,k}),l_{i,k}\}_{k=1}^{n_b(i)}$. At this stage, the algorithm selects the next design configurations and levels of fidelity $\mathscr {B}_{i+1}^{n_b(i+1)}$ to evaluate through the computation and maximization of our acquisition function $U^{\varvec{\pi }^{*}}_{t}$. Let now indicate with $t=i$ the current step of the optimization and with $t+1$ and $t+2$ the first and the second step ahead, respectively. The first element of $U^{\varvec{\pi }^{*}}_{t}$ is determined using the information extracted from the surrogate model updated at the current state of the system $\mathscr {D}_{t}=\mathscr {D}_{i}$. The second element requires our Monte Carlo technique to compute the nested expectation and maximization, and quantify the informative gains at future iterations. Accordingly, the algorithm samples independently a random variable $Z_{j}$ normally distributed for the $j$-th Monte Carlo realization, and simulates the future optimization scenario through the estimate of the mean $\mu ^{(l)}_{t+1}$ and variance $\sigma ^{2(l)}_{t+1}$ of the surrogate model by the computation of Eqs. (15) and (16). This provides an estimate of the multifidelity acquisition function $\widehat{U}^{\varvec{\pi }^{*}}_{t_j}$ as the expectation taken over the realizations $\{\widehat{U}^{\varvec{\pi }^{*}}_{t_j} \}_{j=1}^{N_{MC}}$ (Eq. 18). Then, the penalized maximization of the acquisition function determines the next batch $\mathscr {B}_{i+1}^{n_b(i+1)}$ of design configurations and the levels of fidelity to be evaluated in parallel at the next iteration. This optimization procedure iterates until a maximum computational budget $B_{i} = B_{max}$ is reached, where $B_{i}$ is the cumulative computational cost adopted until iteration $i$.

**Figure 1:**
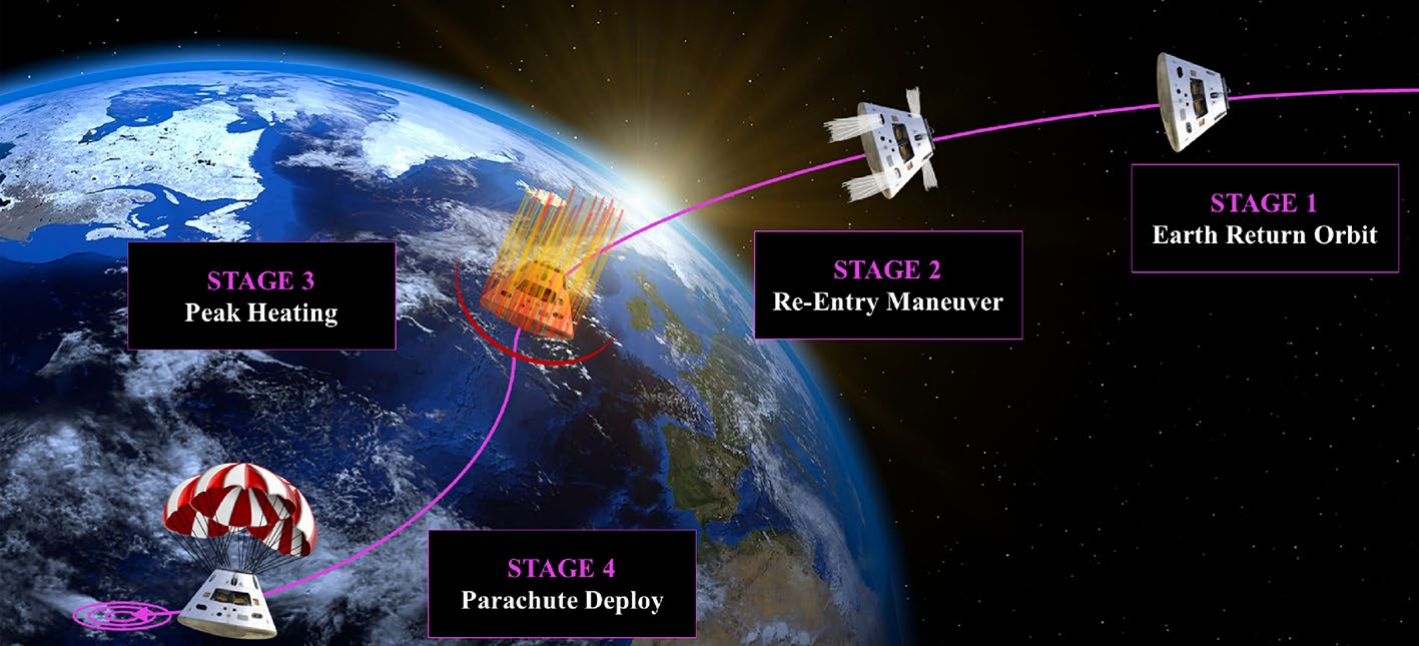
Re-entry mission concept of operations.

## Space vehicle multidisciplinary design optimization

### MDO problem setup

The design of a space re-entry vehicle is a multidisciplinary optimization problem that well carries the computational challenges associated with the design of complex engineering systems^61,62^. This paper uses this demanding MDO applications to demonstrate our NM3-BO scheme and discuss it in comparison with popular standard MFBO algorithms. The space vehicle MDO problem captures the multi-physics nature of the atmospheric re-entry and involves several disciplinary analyses, namely the contributions of the propulsion system, the re-entry descend trajectory, the aerothermodynamic effects that occurs during the descend path, and the thermo-structural interaction between the re-entry flow-field and the thermal protection system. Figure 1 illustrates the concept of operations of the re-entry mission. This involves several phases, namely a maneuver sequence to introduce a thrust component that shapes the re-entry trajectory, the heat peak along the descent caused by the hypersonic aerothermodynamic phenomena, and the deployment of the parachutes during the landing phase.

**Figure 2:**
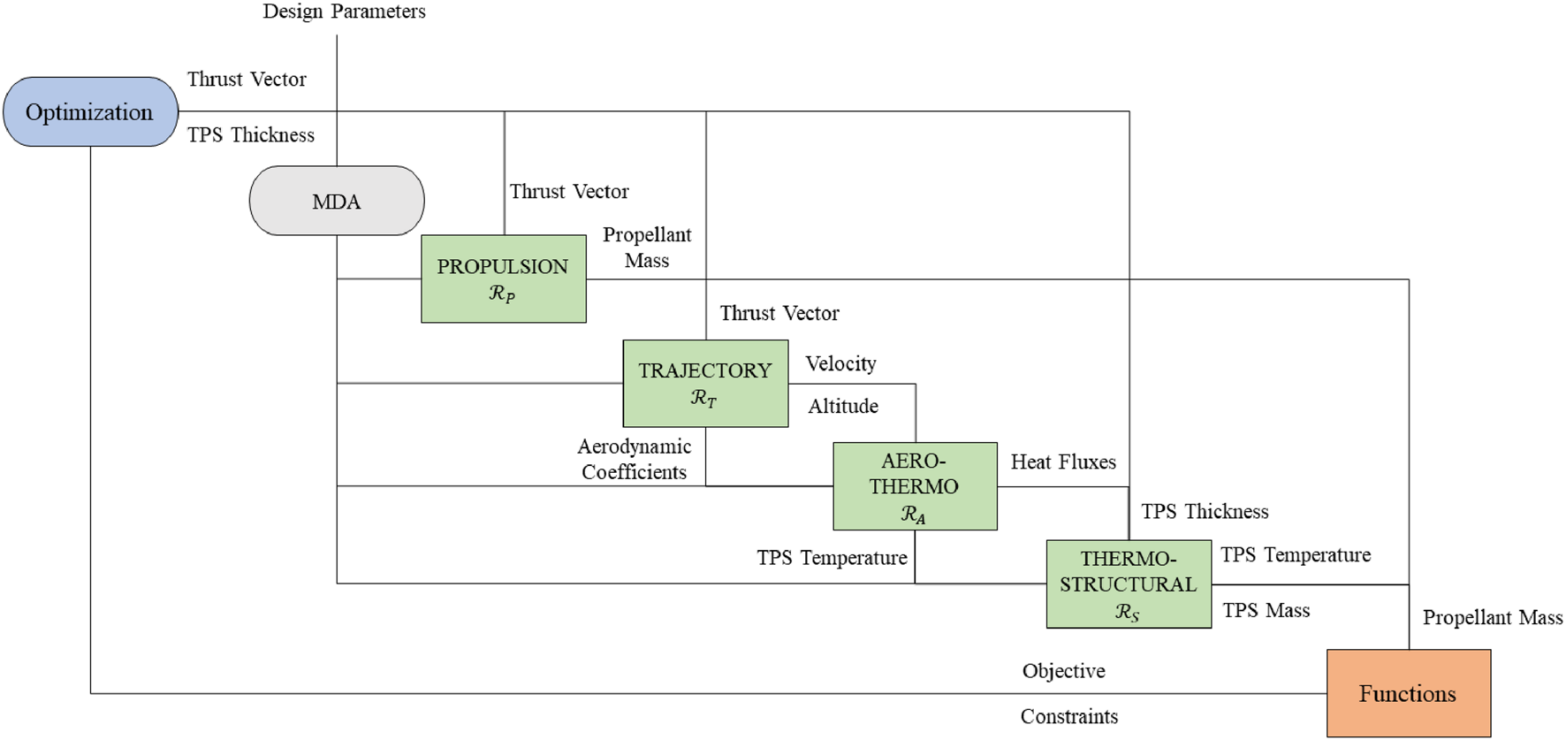
Design structure matrix of the space vehicle MDO problem.

Figure 2 illustrates the design structure matrix^63^ of the re-entry vehicle optimization problem. We adopt the multidisciplinary feasible architecture^4^ to address the MDO problem through a single optimization procedure where the design variables and constraints are under the direct control of the optimizer. The disciplinary analyses flow follows the diagonal of the DSM, while the feed forward flows are represented on the upper triangle and the couplings between disciplines are reported on the lower side. The propulsion system is modeled according to the chemical rocket theory, and comprises primary and secondary chemical thrusters fueled by an hypergolic propellant. The trajectory solver models the descend trajectory as a bi-dimensional orbit propagated through the numerical integration of the non-linear re-entry planetary ordinary differential equations. The aerothermodynamic analysis consists of two disciplinary solvers at different levels of fidelity. The high-fidelity model simulates the full order aerothermodynamic physics through the numerical solution of the Reynolds-Averaged Navier-Stokes equations. The low-fidelity model uses the Oswatitsch Mach number independence principle jointly with the Tauber-Sutton and Sutton-Grave formulations to provide an approximated representation of the aerothermodynamic domain. The high-fidelity model requires hours of computation on an high performance computing cluster, while the low-fidelity analysis is three orders of magnitude faster on a standard computing platform. The thermo-structural analysis models the interaction between the flow-field and the structure of the Thermal Protection System (TPS) through the thermo-elastic equations. Further details about the disciplinary models are provided in the Supplementary Material of this manuscript consistent with what proposed in literature^64^.

The design optimization problem targets the best design configuration ${\textbf {x}}= [F_V, F_N, s_{TPS}]$ of the re-entry vehicle in terms of thrust capabilities $F= [F_V, F_N]$ and TPS structural thickness $s_{TPS}$ that jointly minimizes the temperature $T_{TPS}$ reached by the TPS frame, the overall structural mass $m_{TPS}$ of the TPS, and the mass of propellant $m_{P}$ burned during the re-entry maneuver. The multidisciplinary design optimization problem is formulated as follows:

21 $$\begin{aligned} \begin{aligned} \text {minimize} \quad&f({\textbf {x}}) = 0.4 \frac{m_{TPS}({\textbf {x}})}{m_{TPS0}} + 0.4 \frac{T_{TPS}({\textbf {x}})}{T_{TPS0}} + 0.2 \frac{m_{P}({\textbf {x}})}{m_{P0}} \\ \text {with respect to} \quad&{\textbf {x}}= \left[ F_V, F_N, s_{TPS}\right] \\ \quad \text {subject to} \quad&100 km \le h^{*}({\textbf {x}}) \le 125 km \\ \quad&\mathscr {R}_{P}({\textbf {x}})=0 \\ \quad&\mathscr {R}_{T}({\textbf {x}})=0 \\ \quad&\mathscr {R}^{(l=1)}_{A}({\textbf {x}}) = 0 \\ \quad&\mathscr {R}^{(l=2)}_{A}({\textbf {x}}) \le 10^{-6} \\ \quad&\mathscr {R}_{S}({\textbf {x}})=0 \end{aligned} \end{aligned}$$

where the objectives are evaluated with reference to the baseline values for the TPS mass $m_{TPS0} = 700 \; kg$ and temperature $T_{TPS0} = 1000 \; K$, and for the mass of propellant $m_{P0} = 150 \; kg$ derived from similar re-entry capsules^65^. The search is bounded by the move limits of the design space $\mathscr {X}= \mathscr {X}_{F_V} \times \mathscr {X}_{F_N} \times \mathscr {X}_{s_{TPS}}$, where the thrust capabilities tangential $\mathscr {X}_{F_V} = [29.2 \; kN, 146 \; kN]$ and normal $\mathscr {X}_{F_N} = [0.48 \; kN, 2.4 \; kN]$ to the trajectory are defined according to the propulsion system specifications, and the limits on the TPS thickness $\mathscr {X}_{s_{TPS}} = [0.03 \; m, 0.1 \; m]$ are imposed from expert knowledge. The MDO problem requires a specific range of altitudes $h^{*}$ for the re-entry maneuver to simulate a real-world mission. Additional constraints include the feasibility of the physics-based models at each iteration of the optimization procedure, namely the complete resolution of the propulsion system model $\mathscr {R}_{P}({\textbf {x}}) = 0$, the trajectory model $\mathscr {R}_{T} = 0$, the low-fidelity aerothermodynamic model $\mathscr {R}^{(l=1)}_{A}({\textbf {x}}) = 0$, the high-fidelity aerothermodynamic model ensured reducing the computational residuals below $\mathscr {R}^{(l=2)}_{A}({\textbf {x}}) \le 10^{-6}$, and the thermo-structural model $\mathscr {R}_{S}({\textbf {x}})=0$.

### Results and discussion

The capabilities of the proposed non-myopic multipoint multifidelity algorithm NM3-BO are compared with standard Multifidelity Bayesian Optimization frameworks. All those MFBO algorithms rely on the multifidelity Gaussian process surrogate model and implement different formulations of the acquisition function, including the Multifidelity Expected Improvement^31^ (MFEI), Multifidelity Max-value Entropy Search^32^ (MFMES), and Multifidelity Probability of Improvement^33^ (MFPI). In addition, we report the outcomes achieved with the Efficient Global Optimization^66^ (EGO) algorithm using only high-fidelity queries to provide a comparison with a popular single fidelity Bayesian optimization methodology.

The performance of the competing algorithms are evaluated in terms of the minimum of the objective function $f^*({\textbf {x}}^*) = \min _{{\textbf {x}}\in \mathscr {X}} f({\textbf {x}})$ as a function of the computational budget $B= \sum \lambda _{i}^{(l)}$ at each iteration $i$ of the optimization procedure. The computational costs for the aerothermodynamic analyses are imposed at $\lambda ^{(2)} = 1$ for the high-fidelity model and $\lambda ^{(1)} = 0.001$ for the low-fidelity model; these specific values reflect the time required to complete the aerothermodynamic simulation adopting either the CFD solver or the low-fidelity formulations. We consider a statistics over 25 experiments for each algorithm, and initialize the searches with random initial samples collected through a Latin hypercube sampling scheme. This experimental methodology permits to quantify the influence of different initialization on the algorithms performance. In particular, the multifidelity algorithms are initialized with 1000 design configurations evaluated with the low-fidelity model, and 34 design points computed with the high-fidelity analysis. The single-fidelity algorithm starts the search with an initial set of 35 designs evaluated with the high-fidelity model.

**Figure 3:**
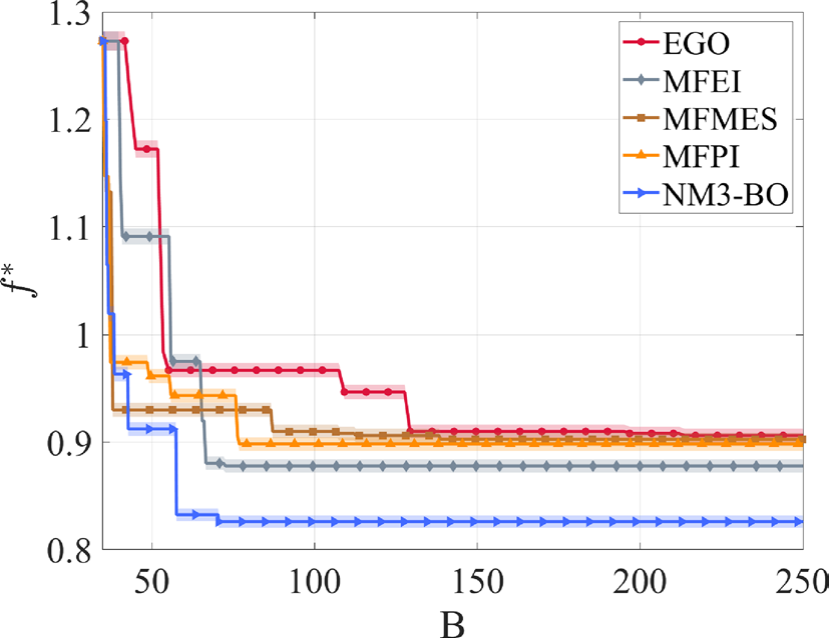
Statistics over 25 experiments of the minimum of the objective function $f^*$ obtained with the competing algorithms.

Figure 3 illustrates the median values (solid line) of the minimum of the objective function $f^*$ along with the observations falling between the 25-th and 75-th percentiles (shaded area). All the initial samples correspond to design configurations that score worse than the baseline solution ($f> 1$), and all the competing algorithms improve the baseline design solution within the maximum computational budget available $B= 250$. A significant achievement is that the multifidelity frameworks outperform the single-fidelity EGO algorithm. This indicates that the combination of data from multiple information sources allows to efficiently explore the design space and contain the computational cost. However, our NM3-BO algorithm obtains superior design solutions – larger reductions of the objective function – with a fraction of the computational cost required by the competing algorithms to identify suboptimal designs. This outcome suggests that the combination of the non-myopic scheme and the multiple decision making process capitalizes from the design evaluations adopting different sources of information, and effectively accelerates the search toward optimal design solutions.

**Table 1:** Median values of the minimum of the objective function $f^*$ and corresponding design improvement $(\cdot )$ obtained with the competing algorithms.

$B$	$f^*_{EGO}$	$f^*_{MFEI}$	$f^*_{MFMES}$	$f^*_{MFPI}$	$f^*_{NM3-BO}$
50	1.1723 (– 17.2 %)	1.0910 (– 9.10 %)	0.9301 (6.99 %)	0.9616 (3.84 %)	0.9123 (8.77 %)
75	0.9670 (3.30 %)	0.8779 (12.21 %)	0.9301 (6.99 %)	0.9434 (5.66 %)	0.8260 (17.4 %)
100	0.9670 (3.30 %)	0.8779 (12.21 %)	0.9101 (8.99 %)	0.8984 (10.16 %)	0.8260 (17.4 %)
150	0.9101 (8.99 %)	0.8779 (12.21 %)	0.9027 (9.73 %)	0.8984 (10.16 %)	0.8260 (17.4 %)
200	0.9082 (9.18 %)	0.8779 (12.21 %)	0.9026 (9.73 %)	0.8984 (10.16 %)	0.8260 (17.4 %)
250	0.9062 (9.38 %)	0.8779 (12.21 %)	0.9026 (9.73 %)	0.8984 (10.16 %)	0.8260 (17.4 %)

**Table 2:** Comparison between the best design solutions identified with the competing algorithms.

Method	$f^*({\textbf {x}}^*)$	${\textbf {x}}^* = [ F_V^*, F_N^*, s_{TPS}^* ]$	$m^*_{TPS}$	$T^*_{TPS}$	$m^*_{P}$
EGO	0.8999 (10.01 %)	${\textbf {x}}^* = [ 33.63 \; kN, 0.969 \; kN, 0.0396 \; m ]$	$476.6 \;kg$	$1320 \; K$	$74.61 \; kg$
MFEI	0.8717 (12.83 %)	${\textbf {x}}^* = [ 35.67 \; kN, 1.561 \; kN, 0.0341 \; m ]$	$410.35 \; kg$	$1326 \; K$	$80.06 \; kg$
MFMES	0.8963 (10.37 %)	${\textbf {x}}^* = [ 35.97 \; kN, 2.046 \; kN, 0.0373 \; m ]$	$447.96 \; kg$	$1329 \; K$	$81.52 \; kg$
MFPI	0.8921 (10.79 %)	${\textbf {x}}^* = [ 35.40 \; kN, 0.691 \; kN, 0.0377 \; m ]$	$453.4 \; kg$	$1322 \; K$	$77.97 \; kg$
NM3-BO	0.8202 (17.98 %)	${\textbf {x}}^* = [ 29.53 \; kN, 0.807 \; kN, 0.0304 \; m ]$	$365.17 \; kg$	$1310 \; K$	$65.45 \; kg$

**Figure 4:**
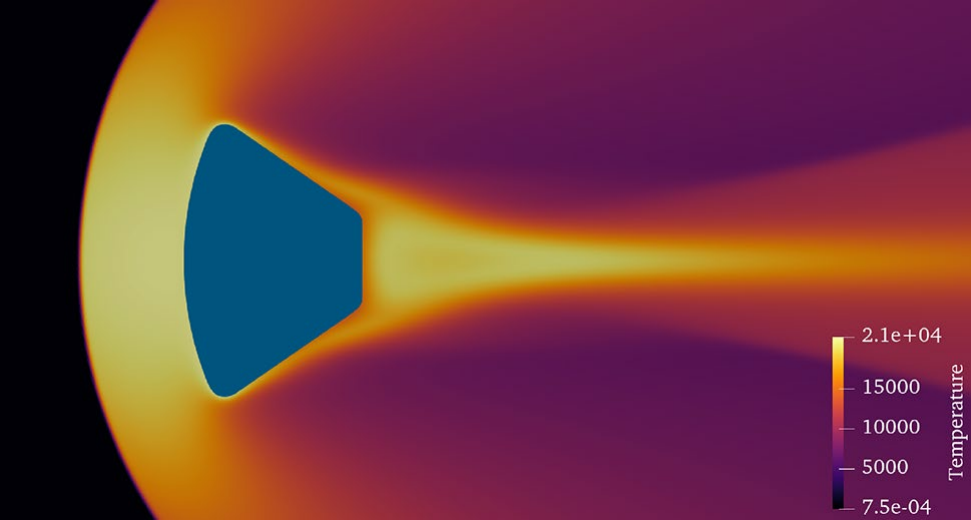
Temperature contours at the heat peak condition evaluated with the high-fidelity aerothermodynamic model considering the best design solution achieved with our NM3-BO algorithm.

To clarify the results from the statistics, Table 1 summarizes the median values of $f^*$ for discrete values of the computational budget $B$. We can observe that the NM3-BO achieves the higher design improvement ($8.77 \%$) after the consumption of a budget $B= 50$, whereas the other strategies score worst in terms of design upgrades. A remarkable outcome is the overall acceleration of the MDO procedure provided by NM3-BO: our framework converges for a computational budget below $B= 75$ and leads to a design upgrade of the $17.4\%$. This result is outstanding if compared with the design improvement of about the $10 \%$ obtained by the EGO, MFMES, and MFPI algorithms, and the design upgrade around the $12 \%$ achieved by the MFEI at convergence.

Table 2 compares the best design solutions obtained with the competing algorithms over the collected experiments. NM3-BO identifies an optimal design configuration of the re-entry vehicle that delivers an upgrade of the $17.98 \%$, and privileges lower thrust capabilities and contained thickness of the thermal protection system. This determines a lower storage of propellant $m^*_{P} = 65.45 \; kg$ on-board and permits to navigate a safe re-entry trajectory that contains the heat loads affecting the frame. As a result, the temperature of the TPS structure is kept below $T^*_{TPS} = 1310 \; K$ with a total TPS structural mass of $m^*_{TPS} = 365.17 \; kg$. Figure 4 provides details about the temperature distribution achieved adopting the best re-entry capsule computed with the NM3-BO at the heat peak condition. It should be noticed that all the design solutions identified by the algorithms prioritize the reduction of both the TPS and propellant mass, and penalize the temperature reached by the heat shield. On one hand, this permits to contain the overall mass of the vehicle with consequent savings in terms of launch costs; on the other hand, the temperature peaks experienced by the structural frame are far below the thermal properties of the TPS material: this guarantees the survival of the vehicle during the atmospheric descent.

## Conclusions

This paper proposes a Non-Myopic Multipoint Multifidelity Bayesian Optimization (NM3-BO) framework to significantly accelerate expensive multidisciplinary design optimization problems. NM3-BO combines two distinguishing features: i) a non-myopic decision making process maximizes the cumulative reward of design solutions over future iterations and ii) a penalization strategy enables multiple decisions as a batch of design configurations and associated level of fidelity to evaluate simultaneously. This search scheme identifies promising batches through the measure of their future utility, and leverages parallel computations to reduce the overall computational cost of the MDO procedure. The NM3-BO algorithm is demonstrated for the MDO problem of a space re-entry vehicle. The method permits substantial accelerations and identifies superior design solutions compared to state-of-the-art multifidelity and single-fidelity algorithms. In particular, NM3-BO delivers on average a space vehicle design improvement of the $17.4 \%$ with a fraction of the computational resources adopted by competing algorithms to identify suboptimal solutions. The results suggest that the non-myopic multiple decision making scheme can pave the way to major computational and energy efficiency gain for the multidisciplinary design of complex engineering systems.

### Supplementary Information

Supplementary Information.

## Data Availability

The datasets generated and analysed during the current study available from the corresponding author on reasonable request.
