# Publisher Correction: Non-myopic multipoint multifidelity Bayesian framework for multidisciplinary design

**DOI:** 10.1038/s41598-024-61882-x

**Published:** 2024-05-14

**Authors:** Francesco Di Fiore, Laura Mainini

**Affiliations:** 1 Departement of Mechanical and Aerospace Engineering, Politecnico di Torino, 10129 Turin, Italy; 2 Department of Aeronautics, Imperial College London, London, SW7 2AZ UK

Correction to: *Scientific Reports* 10.1038/s41598-023-48757-3, published online 18 December 2023

In the original version of this Article, Francesco Di Fiore was incorrectly listed as the corresponding author. The correct corresponding author for this Article is Laura Mainini. Correspondence and request for materials should be addressed to l.mainini@imperial.ac.uk.

The original Article has been corrected.

