# Supplementary material for: Non-myopic multipoint multifidelity Bayesian framework for multidisciplinary design
Source: Sci Rep. 2023 Dec 18;13:22531. doi: 10.1038/s41598-023-48757-3 (PMC10728184; doi:10.1038/s41598-023-48757-3)
Supplement: Supplementary file 1 — Supplementary Information. [file 41598_2023_48757_MOESM1_ESM.pdf]

Space Vehicle Physics-Based Disciplinary Models

Francesco Di Fiore[1] **and Laura Mainini**[2,1,*]

[1]Politecnico di Torino, Departement of Mechanical and Aerospace Engineering, Turin, 10129, Italy
[2]Imperial College London, Department of Aeronautics, London, SW7 2AZ, United Kingdom
[*]l.mainini@imperial.ac.uk

ABSTRACT

This supplementary material details the physics-based disciplinary analysis involved in the multidisciplinary design optimization problem of a space re-entry vehicle. Specifically, the main features and associated governing equations are illustrated and described for the propulsion system, re-entry trajectory, aerothermodynamic couplings, and thermo-structural interactions.

Propulsion System Model

The model of the propulsion system permits to estimate the mass of propellant m_P burned during the re-entry maneuver, given in input the chemical engines specifications summarized in Table 1. Following the chemical rocket theory, the mass of propellant required to complete a maneuver demanding for a thrust magnitude $F = |\boldsymbol{F}|$ can be computed as follows (1):

$$m_P = \int_{t_{on}}^{t_{off}} \frac{F}{c} dt = \frac{F}{c} \Delta t \tag{1}$$

where the thrust vector $\boldsymbol{F} = [F_V, F_N]$ is given by the components tangential F_V and normal F_N to the descend orbit, c is the effective exhaust velocity, t_{on} is the initial time of the maneuver, t_{off} corresponds to the time when the maneuver is completed, and $\Delta = t_{off} - t_{on}$.

Table 1. Propulsion System Design Parameters.

Thrusters Parameters	Description	Unit
Number of primary thruster	2	-
Number of secondary thruster	6	-
Maximum thrust primary thruster (Vacuum)	$F_{max1} = 73$	kN
Maximum thrust secondary thruster (Vacuum)	$F_{max2} = 4.87$	kN
Effective exhaust velocity primary thruster	$c_1 = 2305$	m/s
Effective exhaust velocity secondary thruster	$c_2 = 2943$	m/s
Burning time	$\Delta t = 5$	s

Trajectory Model

The trajectory model computes the descend orbit determined by the re-entry maneuver $\boldsymbol{F}$. This disciplinary analysis requires in input the aerodynamic coefficients that characterize the re-entry vehicle computed through either the high-fidelity or the low-fidelity aerothermodynamic model, and the trajectory design parameters reported in Table 2.

The re-entry profile in terms of descend velocity V, flight path angle γ, altitude during the re-entry h, and longitude angle β is obtained considering a planar trajectory assuming the Earth with negligible rotation and constant flight path azimuth angle:

$$\frac{dV}{dt} = -\frac{(D+F_V)}{M} - g\sin\gamma \tag{2a}$$

$$V\frac{d\gamma}{dt} = \frac{(L+F_N)}{M}L - g\cos\gamma + \frac{V^2}{(h+R_E)}\cos\gamma \tag{2b}$$

$$\frac{dh}{dt} = V\sin\gamma \tag{2c}$$

$$\frac{d\beta}{dt} = \frac{V\cos\gamma}{(h+R_E)} \tag{2d}$$

where t is the re-entry time, M is the overall mass of the vehicle, D and L are respectively the aerodynamic drag and lift, g is the acceleration of gravity, and R_E is the Earth radius.

The system of non-linear ODEs (Equation (2)) is numerically solved adopting the Runge-Kutta method integrating the equations over the re-entry time. The entry maneuver is considered as impulsive given the contained burning time that characterize the chemical thrusters, allowing to consider the effect of the thrust components F_V and F_N exclusively during the first integration step.

Table 2. Trajectory Design Parameters.

Trajectory Parameters	Description	Unit
Initial velocity	$V_0 = 7900$	m/s
Initial longitude	$\beta_0 = 0$	deg
Initial time	$t_0 = 0$	s
Capsule mass	$M = 7500$	kg
Capsule reference area	$A_{ref} = 78.54$	m^2
Altitude for parachute deployment	$h = 5000$	m

High-Fidelity Aerothermodynamic Model

The high-fidelity aerothermodynamic model computes the heat flux $\dot{q}$ at the stagnation point of the thermal protection system and the aerodynamic coefficients of lift C_L and drag C_D. This model requires in input the geometric features of the re-entry vehicle (Table 3) together with the re-entry profile in output from the trajectory model and the temperature of the heat shield T_{TPS} computed with the thermo-structural model.

The flow-field experienced by the vehicle during the re-entry is modelled through the full set of Reynolds-Averaged Navier-Stokes (RANS) equations to account for the effects of turbulence and unsteadiness. We use the finite volume method to discretize the RANS equations in space, adopting a standard edge-based data structure where the convective and viscous fluxes are computed at the midpoint of the edges. The fluid domain is geometrically defined as a semicircle of radius $6.3R_N$ to avoid shock reflections, and is discretized with a total of $9.2 \cdot 10^4$ quads elements. The computational grid is characterizes by a refined density of the mesh in proximity of the thermal protection system of the vehicle, to accurately capture the severe aerothermodynamic phenomena critical for the structural frame. The RANS equations are numerically solved through SU2 (2) version 7.0.3 computational fluid dynamic solver, and the computational grid is generated adopting the gmsh software (3). The governing equations are integrated through the Euler implicit scheme, where the convergence criteria is set for computational residuals 10^{-6}.

Low-Fidelity Aerothermodynamic Model

The low-fidelity aerothermodynamic model provides a fast approximation of the aerodynamic coefficients C_L and C_D of the vehicle and the stagnation heat flux $\dot{q}$ affecting the TPS frame, given the re-entry profile and the geometry of the capsule (Table 3). This representation uses two physics-based surrogate models that allow to evaluate the aerothermodynamic phenomena with a fraction of the computational cost required to compute the high-fidelity model. Specifically, the aerodynamic coefficients are approximated through the Oswatitsch Mach number independence principle (4), and the stagnation heat flux is computed as the sum of convective and radiative effects adopting the Sutton-Grave (5) and Tauber-Sutton (6) formulations.

Table 3. Geometry Design Paramaters of the Re-Entry Vehicle.

Geometry Parameters	Description	Unit
Frontal section diameter	$R = 5.0$	m
Nose radius	$R_N = 2.4R$	m
Upperside ablator inclination	$\theta_A = 32.5$	deg
TPS aperture	$\theta_D = 23.04$	deg

The Mach number independence principle defines the aerodynamic coefficients constant with altitude assuming that the flow-field is governed by inviscid Euler equations; this implies that the flow-field tends to a limit condition at high values of the Mach number that characterizes the re-entry trajectory, for which C_L and C_D assume constant limit values.

The total heat flux at the stagnation point is computed as follows:

$$\dot{q} = \dot{q}_{conv} + \dot{q}_{rad} \tag{3}$$

The convective heat flux is evaluated following the Sutton-Grave formulation (5):

$$\dot{q}_{conv} = k_s \sqrt{\frac{\rho_\infty}{R_N}} \left(\frac{V}{1000}\right)^{3.15} \tag{4}$$

where $k_s = 5.1564 \cdot 10^{-5}$ is a constant for the Earth atmosphere, R_N is the radius of the nose of the capsule and V is the re-entry flight velocity.

The radiative heat flux is estimated according to the Tauber-Sutton formulation (6):

$$\dot{q}_{rad} = C R_N^a \rho_\infty^b f(V) \tag{5}$$

where $C = 4.736 \cdot 10^4$ and $b = 1.22$ are constants for the Earth atmosphere, $a = 1.072 \cdot 10^6 V^{-1.88} \rho_\infty^{-0.325}$ is given in function of the descend velocity V and the density of the atmosphere $\rho_\infty(h)$, and $f(V)$ is a tabulated function of velocity.

Thermo-Structural Model

The thermo-structural representation models the interactions between the flow field heat fluxes affecting the structure and the frame of the thermal protection system. This model evaluates the temperature of the TPS structure T_{TPS} and the mass of the TPS frame m_{TPS}, given the total heat load $\dot{q}$ provided by the high-fidelity or low-fidelity aerothermodynamic analysis, the thickness of the TPS structure s_{TPS}, the geometry of the capsule (Table 3), and the material property of the TPS (Table 4).

The thermo-structural interaction is evaluated adopting the finite element method to numerically approximate the governing heat equation. In particular, the TPS frame is represented as an arc of circumference discretized with $n_e = 1000$ linear elements; this permits to formulate the heat equation for the generic e-th finite element considering a linear formulation where the thermal conductivity κ_{TPS} and the thickness of the thermal protection system s_{TPS} are assumed uniform:

Table 4. Design parameters of the thermal protection system.

TPS Parameter	Description	Unit
TPS mass density	$\rho_{TPS} = 6000$	kg/m^3
TPS specific heat (constant pressure)	$c_P = 628$	J/kgK
TPS emissivity	$\varepsilon_{TPS} = 0.9$	$-$
TPS thermal conductivity ($T = 300K$)	$\kappa_{TPS} = 58$	W/mK
TPS thermal conductivity ($T = 1300K$)	$\kappa_{TPS} = 64$	W/mK
TPS thermal conductivity ($T = 2300K$)	$\kappa_{TPS} = 134$	W/mK
TPS maximum temperature	$T_{max} = 2273.15$	K

$$\rho_{TPS} c_{PS} s_{TPS} \frac{dT}{dt} - \kappa_{TPS} s_{TPS} \frac{\partial^2 T}{\partial \eta^2} + 4\sigma \varepsilon_{TPS} T_\infty^3 T - 4\sigma \varepsilon_{TPS} T_\infty^4 - \dot{q}_s = 0 \tag{6}$$

where ρ_{TPS}, c_P and ε_{TPS} are respectively the density, the specific heat at constant pressure and the emissivity coefficient of the TPS material, σ is the Stephan-Boltzmann constant, $\dot{q}_s$ is the heat source term and $T_\infty(h)$ is the temperature of the atmosphere. The numerical solution of Equation (6) is computed through the Galerkin method over the discretized domain that models the structure of the TPS.

In addition, the thermo-structural model estimates the total mass of the structural frame of the TPS m_{TPS} as a function of the thickness s_{TPS}:

$$m_{TPS} = \rho_{TPS} S_{TPS} s_{TPS} \tag{7}$$

where S_{TPS} is the frontal surface of the structure of the TPS which corresponds to the circular are with radius equal to the radius of the nose of the capsule R_N.

References

1. Sutton, G. P. & Biblarz, O. *Rocket propulsion elements* (John Wiley & Sons, 2016).

2. Palacios, F. *et al.* Stanford university unstructured (su2): An open-source integrated computational environment for multi-physics simulation and design. *AIAA paper* **287**, 2013 (2013).

3. Geuzaine, C. & Remacle, J.-F. Gmsh: A 3-d finite element mesh generator with built-in pre-and post-processing facilities. *Int. journal for numerical methods engineering* **79**, 1309–1331 (2009).

4. Oswatitsch, K. Ähnlichkeitsgesetze für hyperschallströmung. *Zeitschrift für angewandte Math. und Physik ZAMP* **2**, 249–264 (1951).

5. Sutton, K. & Graves Jr, R. A. A general stagnation-point convective-heating equation for arbitrary gas mixtures. *Work* **50**, 7885 (1971).

6. Tauber, M. E. & Sutton, K. Stagnation-point radiative heating relations for earth and mars entries. *J. Spacecr. Rocket.* **28**, 40–42 (1991).
